# Diabetic Cardiomyopathy: Does the Type of Diabetes Matter?

**DOI:** 10.3390/ijms17122136

**Published:** 2016-12-18

**Authors:** Maximilian E. Hölscher, Christoph Bode, Heiko Bugger

**Affiliations:** Cardiology and Angiology I, University Heart Center Freiburg, Hugstetter Str. 55, 79106 Freiburg, Germany; maximilian.hoelscher@universitaets-herzzentrum.de (M.E.H.); christoph.bode@universitaets-herzzentrum.de (C.B.)

**Keywords:** diabetic cardiomyopathy, heart, diabetes mellitus

## Abstract

In recent years, type 2 diabetes mellitus has evolved as a rapidly increasing epidemic that parallels the increased prevalence of obesity and which markedly increases the risk of cardiovascular disease across the globe. While ischemic heart disease represents the major cause of death in diabetic subjects, diabetic cardiomyopathy (DC) summarizes adverse effects of diabetes mellitus on the heart that are independent of coronary artery disease (CAD) and hypertension. DC increases the risk of heart failure (HF) and may lead to both heart failure with preserved ejection fraction (HFpEF) and reduced ejection fraction (HFrEF). Numerous molecular mechanisms have been proposed to underlie DC that partially overlap with mechanisms believed to contribute to heart failure. Nevertheless, the existence of DC remains a topic of controversy, although the clinical relevance of DC is increasingly recognized by scientists and clinicians. In addition, relatively little attention has been attributed to the fact that both underlying mechanisms and clinical features of DC may be partially distinct in type 1 versus type 2 diabetes. In the following review, we will discuss clinical and preclinical literature on the existence of human DC in the context of the two different types of diabetes mellitus.

## 1. Introduction

Diabetes mellitus has become a rapidly growing epidemic in recent decades. Diabetic complications remain the main cause of morbidity and mortality in diabetic patients, with cardiovascular disease being the leading cause of death. While coronary artery disease (CAD) is the main cause of heart failure (HF) and cardiovascular death in diabetics, the risk of heart failure remains increased despite adjustment for CAD and hypertension [1]. Thus, the term “diabetic cardiomyopathy” (DC) was introduced to refer to this cardiac entity, defined as ventricular dysfunction in the absence of CAD and hypertension [2]. Clinically, DC is characterized by cardiac hypertrophy and diastolic dysfunction which may result in heart failure with preserved ejection fraction (HFpEF) [3]. Some authors even argue that DC may result in systolic heart failure (HFrEF) although convincing evidence from prospective studies is still lacking [3]. For a few years, DC has been increasingly recognized by clinicians, underpinned by the increasing number of publications on DC. However, since DC mostly lacks classical features of a cardiomyopathy in clinical studies, such as ventricular dilation and meaningful systolic dysfunction, the debate about the existence of DC remains controversial. Instead of being a cardiomyopathy in the classical sense though, DC rather represents a combination of molecular myocardial abnormalities that predispose for the development of myocardial dysfunction, in particular in the presence of additional stressors such as hypertension and CAD. In the following paragraphs, we will discuss clinical and preclinical literature on the existence of human DC, including a discussion whether type 1 and type 2 diabetes may cause distinct types of DC.

## 2. Diabetic Cardiomyopathy in Type 2 Diabetes?

Several studies reported an increased risk of heart failure in patients with type 2 diabetes. The U.K. Prospective Diabetes Study (UKPDS) with 4585 patients showed that an increase in HbA_1c_ was associated with a higher risk of heart failure incidence (2.3–11.9 per 1000 patient-years) over 10 years of follow-up [4]. Nichols et al. showed that patients with diabetes developed congestive heart failure in 30.9 cases per 1000 person-years compared to 12.4 cases in non-diabetic counterparts, thus reflecting a 2.5-fold increase in heart failure risk in diabetics. When comparing patients that develop heart failure, patients with diabetes were on average 5.5 years younger than non-diabetic subjects. The difference in the rates of HF development between non-diabetic and diabetic subjects turned out to be much greater in younger-aged groups [5]. The increased risk of heart failure is however not completely explained by major risk factors for heart failure such as hypertension or CAD. In fact, Iribarren et al. reported an increase in the incidence of heart failure with increasing HbA_1c_ in a monotonic fashion in a cohort of 48,858 patients with diabetes. But importantly, adjustment for race, level of education, cigarette smoking, alcohol consumption, hypertension, obesity, ACE inhibitors, beta-blockers, diabetes type and duration, and interim myocardial infarction attenuated but did not completely explain the association [6]. Similarly, Kannel and colleagues also reported an increased risk of HF in type 2 diabetic subjects despite adjustment for CAD, hypertension, age, sex, cholesterol and body weight already decades ago [7]. Other important findings supporting the existence of a DC in type 2 diabetes include data on myocardial infarct size and function. Jaffe et al. reported a higher incidence of chronic HF in patients with diabetes despite a smaller infarct size compared to non-diabetic patients, suggesting pathogenetic processes that are additional to CAD in the diabetic heart such as impaired reflex adaption to hemodynamic stress, autonomic dysfunction or an occult diabetic cardiomyopathy [8]. Iwasaka and colleagues showed that the regional ejection fraction of the non-infarcted area following myocardial infarction was decreased in type 2 diabetic patients although global ejection fraction was not reduced, suggesting that the non-infarcted area was unable to compensate for the loss of function in the infarcted area [9]. Taken together, the above mentioned studies strongly suggest that there is an increased myocardial vulnerability to develop cardiac dysfunction and heart failure in type 2 diabetes.

The clinical features of DC in type 2 diabetes may include cardiac hypertrophy and diastolic dysfunction. Eguchi et al. showed that there is a positive relationship between type 2 diabetes and an increased left ventricular mass, independent of obesity, race/ethnicity, physical activity and, in addition, they found a weaker association with the use of antihypertensive medication [10]. Consistent with these results, Devereux et al. detected an association of increased left ventricular (LV) absolute and relative wall thickness as well as LV mass with type 2 diabetes. Compared to non-diabetic patients, LV mass index was increased by 6%–9% and by 12%–14% in diabetic women and men, respectively [11]. Echocardiographic data from the original Framingham Study cohort and the Framingham Offspring Study showed increased LV wall thickness, enddiastolic dimension and LV mass in diabetic women. Interestingly, there was no difference in LV wall thickness in male subjects, although fractional shortening was slightly reduced [12,13]. Regarding diastolic dysfunction, Fontes-Carvalho et al. described a progressive worsening of diastolic function parameters such as E′ velocity and E/E′ ratio in individuals with type 2 diabetes compared to patients without diabetes. Impairment in diastolic function was independent of age, blood pressure and body mass index (BMI) [14]. Similarly, Stahrenberg et al. showed that the prevalence and severity of diastolic dysfunction increased significantly along the diabetic continuum. Echocardiographic parameters such as E/E′ were similar in patients with prediabetes compared to those with normal glucose metabolism but became significantly higher in patients with type 2 diabetes, emphasizing a correlation of worsening diastolic function with increasing HbA_1c_ [15]. Both LV hypertrophy and diastolic dysfunction may increase the risk of pulmonary congestion and edema, which may lead to HF with preserved ejection fraction (HFpEF), a complex disease which may account for up to 50% of all HF cases in the general population and which remains a treatment challenge for cardiologists. The risk of the development of HFpEF in type 2 diabetics may be particularly increased if additional stressors are present in diabetic subjects, such as ischemic cardiomyopathy, hypertension or impaired vascular dynamics. Convincing evidence of a meaningful impairment in systolic function in DC of type 2 diabetic patients is lacking to date. Interestingly though, recent studies using echocardiographic strain analysis reported of subclinical impairment in systolic function, including impairment in longitudinal and radial strain [16]. These studies may suggest that the molecular alterations in DC in type 2 diabetics, which do largely overlap with mechanisms in heart failure, may indeed cause intrinsic defects in contractile function [17]. More studies are needed that address the potential defects in systolic function due to DC in type 2 diabetic patients. Notably, there is also a well-established impairment of myocardial flow reserve in DC which may contribute to systolic dysfunction, likely related to a microvascular dysfunction as a consequence of perivascular and interstitial fibrosis, reduced capillary density, and endothelial dysfunction [18,19]. In addition, recent studies demonstrated an association of impaired coronary flow reserve with LV filling pressure in patients with type 2 diabetes, suggestive of a possible link between coronary microvascular disease and diastolic dysfunction in type 2 diabetes [20]. Thus, systolic and diastolic dysfunction may be related to a small vessel disease, as opposed to or in addition to direct damage of the diabetic milieu on cardiomyocyte physiology.

While evidence of the existence of a DC in type 2 diabetes is steadily increasing, a major criticism still remains the lack of a prospective clinical trial clearly showing an increased risk of HF and/or impaired cardiac function in the complete absence of confounders, e.g., CAD and hypertension. However, such a study would be costly, would require a long-term follow-up period, and would require a large amount of patients since many diabetic patients will develop confounding comorbidities during the course of such a trial. Other critiques include that the changes in diastolic function may be attributable to an increase in peripheral resistance and myocardial overload mediated by hyperinsulinemia and secondary activation of the sympathetic nervous system. Insulin increases cell growth, thus it has been suggested that cardiac hypertrophy in type 2 diabetes may result from hyperinsulinemia [21]. Some authors also argue that subclinical systolic dysfunction detectable by strain imaging may also be observed in many other conditions, including obesity, hypertension, and even healthy aging, and thus may not be specific to diabetes.

## 3. Diabetic Cardiomyopathy in Type 1 Diabetes?

Only a few studies have examined the risk of development of heart failure in type 1 diabetic patients. Lind et al. showed an increased risk of developing heart failure in type 1 diabetic patients. In a large study performed with data from the Swedish national diabetes registry, the incidence of HF per 1000 patient-years was found to increase monotonically and inversely with glycemic control. After adjustment for age, sex and diabetes duration, the hazard ratio for the highest category of HbA_1c_ (>10.5%) with the lowest category of HbA_1c_ (<6.5%) as a reference was 6.4. Even after further adjustment for smoking status, blood pressure, BMI and comorbidities such as myocardial infarction, the risk of heart failure in patients with very poor glycemic control was four times that in patients with optimum glycemic control. Each increase of 1% in HbA_1c_ was associated with a 30% higher risk of HF, independently of other risk factors such as high blood pressure, diabetes, smoking and BMI. During a 9-year follow-up period, hospital admission for HF occurred in almost one out of 30 young patients with type 1 diabetes, implying that HF was a major diabetic complication in these patients [22]. In another study, the incidence rate of HF was 2.1 per 1000 person-years in a random population at the age of 55–64 years [23], whereas the incidence of HF in a group of men aged 41–45 years with type 1 diabetes was 2.4 per 1000 patient-years, indicating that the risk of HF in patients with type 1 diabetes is similar to that observed in a general population of people that are 15 years older compared to the diabetic cohort [22]. Compatible with these findings, a registry-based prospective case-control study performed by Rosengren et al. showed that 3% of type 1 diabetic patients were admitted to a hospital over a mean follow-up of 7.9 years with a diagnosis of heart failure compared to 1% of matched controls. The incidence rates for any heart failure diagnosis were 4.0 per 1000 person-years in diabetic patients compared to 0.97 per 1000 person-years in controls [24]. These studies may thus support the conclusion of an increased risk of HF in type 1 diabetic subjects. And this may be the case although patients were treated with insulin, which is a causal treatment in type 1 diabetes and may thus attenuate the chronic impact of systemic metabolic derangements in type 1 diabetics on the heart. Of interest though, a recent study by Konduracka and colleagues observed type 1 diabetic patients over an average of 36 years and demonstrated that the HF incidence was rather low, and HF and myocardial dysfunction were only observed if patients developed hypertension or CAD [25]. Despite the presence of microvascular dysfunction and fibrosis, no significant echocardiographic differences were observed in long-term type 1 diabetes in another study [21].

The impact of type 1 diabetes on systolic and diastolic function is less clear compared to type 2 diabetes. Raev et al. showed that diastolic dysfunction is more common than systolic dysfunction in subjects with type 1 diabetes, and that myocardial damage in type 1 diabetes may impair diastolic function before systolic function [26]. It has been shown that type 1 diabetes negatively impacts echocardiographic parameters of diastolic function, in particular the E/A ratio (ratio of early diastolic filling/late diastolicfilling of the left ventricle) and transmitral blood flow velocity, as a sign of premature aging of the heart. The values measured in 20–32 year-old persons with type 1 diabetes corresponded with data obtained from healthy men at the age of 50 years, indicating an early onset of diastolic dysfunction in type 1 diabetes compared to a control population [27]. These findings were supported by a study performed on 8–18 year-old children and young adolescents with a mean diabetes duration of 5.6 years who showed reduced diastolic function with higher peak a′-velocity and reduced e′/a′-ratio, indicating impaired diastolic function after a short duration of disease. The e′/a′ ratio was negatively associated with increased blood pressure and body mass index but not with sex, diabetes duration, vessel elasticity or HbA_1c_ levels [21]. In contrast to diastolic function, LV systolic function was not impaired in most studies [26,28], while one study even reported increased systolic function in type 1 diabetic patients [29,30] however, demonstrated an early impairment of LV systolic function at rest by using color doppler myocardial imaging (reduction of systolic strain and systolic strain rate) and integrated backscatter imaging (reduction of the cyclic variation index) while LV systolic function expressed by conventional indices such as fractional shortening and ejection fraction did not show any differences. A lack of diastolic dysfunction in this study was explained by the authors in a more strict selection of patients with good glycemic control and a short duration of diabetes. Labombarda et al. demonstrated that diabetes in young patients is associated with an alteration of longitudinal LV deformation with a positive correlation to glycemic control. Impairment in global longitudinal strain was described to be controlled by subendocardial longitudinal myofibres which are susceptible to ischemia and fibrosis. Thus subclinical impairment of longitudinal strain may represent the first anomaly observed in heart failure [31]. Other studies showed abnormal contractile responses only during exercise and during increased cardiac stress but not at rest [32,33] Finally, some studies showed neither a difference in systolic nor in diastolic function between type 1 diabetic patients and a control group [34]. Thus, the true impact of type 1 diabetes on the heart remains incompletely elucidated in human studies. However, as mentioned above, one should keep in mind that type 1 diabetic patients are usually treated with insulin, which basically normalizes the metabolic derangements induced by insulin deficiency and may thus attenuate the true detrimental effects of type 1 diabetes on the heart.

Compared to type 2 diabetes, there is certainly more variability of data reported in type 1 diabetic patients. This may be related to the fact that patient selection and exclusion criteria may have varied considerably between studies. Whether diastolic dysfunction is due to diabetes per se remains controversial since adjustment for coexistent hypertension, CAD, autonomic dysfunction, and microangiopathy blunted the significance for diastolic dysfunction in some studies [7]. Once again, it is important to consider the fact that the lack of cardiac dysfunction may be related to permanent treatment with exogenous insulin, which is a causal treatment in type 1 diabetes. It has also been argued that myocardial overload and increased peripheral resistance after the administration of exogenous insulin may cause diastolic dysfunction, as opposed to being symptoms of DC. Others discussed that in some studies, echocardiographic parameters of diastolic dysfunction were misinterpreted since absolute values that were significantly different between diabetic and nondiabetic subjects were actually (according to echocardiographic guidelines) within the normal range for healthy people, which would not allow to diagnose diastolic dysfunction in the diabetic cohort [35].

## 4. Preclinical Data on Diabetic Cardiomyopathy

Numerous studies have been performed in animal models of diabetes investigating phenotypes and underlying mechanisms of DC. Particularly in type 2 diabetes models, many findings were subsequently confirmed to occur in human DC as well, indicating that findings in rodents models of diabetes can indeed be extrapolated to the human heart to gain more insight into the pathophysiology of human DC. It is important to note that rodents are somewhat resistant to the development of CAD unless predisposing mutations for the development of atherosclerosis are introduced, making rodents a convenient species to study DC [36,37].

Cardiac hypertrophy evidenced by increased LV mass and LV wall thickness, and diastolic dysfunction measured by echocardiography or MRI, have been consistently observed in various rodent models of type 2 diabetes, including *db*/*db* mice, *ob*/*ob* mice and Zucker diabetic fatty rats [36,38,39,40]. Depending on the degree of hyperglycemia and diabetes, systolic dysfunction is also observed in these models using echocardiography or using ex vivo techniques such as Langendorff heart perfusion or the isolated working heart model [39,41,42]. Numerous mechanisms have been proposed to underlie the development of diabetic cardiomyopathy, many of which overlap with failing hearts, including oxidative stress, mitochondrial dysfunction, increased fibrosis, impairment in calcium handling, increased inflammation, augmented cell death and increased activation of the renin-angiotensin system, to name a few [17]. These pathologic alterations in cardiomyocytes are primarily triggered by systemic metabolic alterations such as hyperglycemia, hyper- and dyslipidemia, hyperinsulinemia and insulin resistance. Increased myocardial fatty acid uptake and generation of toxic lipid intermediates contribute to increased apoptosis, oxidative stress and LV dysfunction, and the cardiac manifestation of insulin resistance may contribute to mitochondrial dysfunction and uncoupling, oxidative stress, cardiac inefficiency, and myocardial energy depletion [43,44,45]. Thus, there is consistent evidence of a DC in rodent models of type 2 diabetes, and many findings have been reproduced in humans, strongly suggesting the existence of a human DC in type 2 diabetes.

Similar to animal models of type 2 diabetes, diastolic dysfunction is also observed in commonly used rodent models of type 1 diabetes [36]. Diastolic dysfunction has been suggested by increased LV diastolic pressure using cardiac catheterization and abnormal patterns of mitral inflow and pulmonary venous flow using Doppler echocardiography in streptozotocin (STZ)-treated rodents and Akita diabetic mice [46,47,48]. Regarding systolic function, impairment in ejection fraction and cardiac dysfunction in isolated heart perfusion models is consistently reported in the STZ model of type 1 diabetes, if duration of diabetes is sufficient [46,49]. Of interest, a recent study assessing early features of DC in STZ mice one week after injection by using cardiac magnetic resonance demonstrated early reductions of LV volumes which may be explained by hypovolemia due to hyperglycemic osmotic diuresis, suggestive of a hemodynamic mechanism contributing to cardiac dysfunction in this model of DC [50]. In OVE26 mice, impaired contractility was only observed in isolated cardiomyocytes but not in the Langendorff heart perfusion [51,52]. In Akita diabetic mice, systolic function was preserved both in vivo and ex vivo at young and old ages [53,54]. Of interest, cardiac hypertrophy is usually not observed, but instead hearts may rather be smaller compared to non-diabetic controls [54,55]. This observation may be related to the lack of insulin`s effect on cellular growth and protein synthesis, as also underlined by decreased cardiac size in mice lacking insulin receptors specifically in cardiomyocytes [44]. Regarding underlying mechanisms of DC, many observations seem to overlap with alterations in hearts of type 2 diabetes, but not in all models and not all abnormalities. For example, Akita diabetic mice do not seem to develop fibrosis, increased myocardial inflammation, oxidative stress or an impairment in cardiac efficiency, although some typical traits of DC are observed, such as impaired calcium handling, mitochondrial dysfunction, or increased fatty acid utilization [48,53,54]. As another example, mice with STZ-induced type 1 diabetes do not show impaired cardiac efficiency, unless cardiomyocyte insulin resistance, a feature of type 2 diabetic hearts, is induced by deletion of cardiomyocyte insulin receptors [55]. For the STZ model, it needs to be kept in mind as well that STZ may exert extrapancreatic toxic effects that may also influence the cardiac phenotype in this model [56]. As in humans, insulin treatment can quickly reverse phenotypes and abnormalities in type 1 diabetic animals, such as diastolic dysfunction, decreased expression of sarcoendoplasmic reticulum Ca^2+^-ATPase 2a (SERCA2a), mitochondrial dysfunction, or oxidative stress [48,57]. Thus, both the phenotype and molecular mechanisms seem to be distinct, at least in part, both among models of type 1 diabetes, and compared to type 2 diabetic models.

## 5. Conclusions

**Figure 1:**
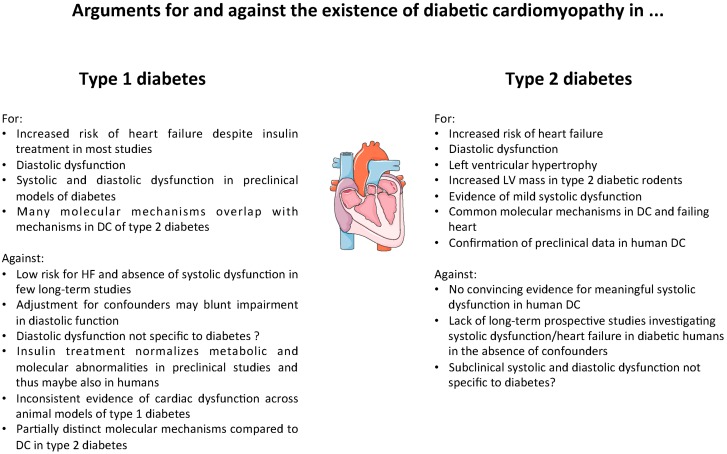
A compilation of arguments for and against the existence of diabetic cardiomyopathy in type 1 and type 2 diabetes. HF, heart failure; DC, diabetic cardiomyopathy; LV, left ventricular.

There is good evidence both in animals and humans that a DC exists in type 2 diabetes that affects cardiac function and morphology and that increases the risk of the development of heart failure (Figure 1). In how far type 1 diabetes affects the heart in the absence of CAD and hypertension remains controversial, mainly since results of human studies are ambiguous and since patients are usually treated with insulin which may attenuate or mask the phenotype. Similarly, not all myocardial abnormalities of type 2 diabetic animals are recapitulated in models of type 1 diabetes. In this respect, it should be noted that systemic metabolic derangements such as hyperglycemia and dyslipidemia are similar in most models of type 1 and type 2 diabetes, thus pointing towards differences in myocardial insulin action (insulin resistance with accompanying hyperinsulinemia versus insulin deficiency) as an underling factor that may explain phenotypic differences between the distinct types of diabetes. In fact, signaling downstream of the insulin receptor is very likely differentially affected in hearts of type 1 and type 2 diabetes as well, which may have deleterious effects on cell growth, cell survival and other cellular pathways [45]. It becomes clear that our understanding of DC, which has been known for more than 40 years, is still preliminary, and it seems likely that type 1 and type 2 diabetes differentially affect the heart, at least in part. Nevertheless, a DC also seems to exist in type 1 diabetic humans, which needs to be characterized in more detail, in particular regarding molecular alterations within the myocardium.

## Abbreviations

DC	Diabetic cardiomyopathy
HF	Heart failure
CAD	Coronary artery disease
HFpEF	Heart failure with preserved ejection fraction
HFrEF	Heart failure with reduced ejection fraction
